# Reducing Oxidative Stress-Mediated Alcoholic Liver Injury by Multiplexed RNAi of *Cyp2e1, Cyp4a10*, and *Cyp4a14*

**DOI:** 10.3390/biomedicines12071505

**Published:** 2024-07-06

**Authors:** Qi Zhang, Shuang Wu, Qiubing Chen, Yahong Zhang, Cai Zhang, Runting Yin, Zhen Ouyang, Yuan Wei

**Affiliations:** 1 School of Pharmacy, Jiangsu University, Zhenjiang 212013, China; zhangqi990212@163.com (Q.Z.); wushuang873@163.com (S.W.);; 2 Department of Urology, Frontier Science Centre for Immunology and Metabolism, Medical Research Institute, Zhongnan Hospital of Wuhan University, Wuhan University, Wuhan 430071, China; 3 Department of Laboratory Medicine, Zhongnan Hospital of Wuhan University, Wuhan 430071, China

**Keywords:** alcoholic liver disease, cytochrome P450s, in vivo RNAi, inflammation, ferroptosis

## Abstract

The prevalence of excessive drinking-related alcoholic liver disease (ALD) is rising, yet therapeutic options remain limited. High alcohol consumption and consequent oxidative metabolism by cytochrome P450 (CYP) can lead to extremely high levels of reactive oxygen species, which overwhelm cellular defenses and harm hepatocytes. Our previous investigations showed that inhibiting *Cyp2e1* using RNA interference reduced the incidence of ALD. However, compensatory mechanisms other than CYP2E1 contribute to oxidative stress in the liver. Therefore, we coupled triple siRNA lipid nanoparticles (LNPs) targeting *Cyp2e1* with two isoenzymes *Cyp4a10* and *Cyp4a14* to treat ALD mouse models fed with Lieber–Decarli ethanol liquid diet for 12 weeks at the early (1st week), middle (5th week), and late (9th week) stages. The administration of triple siRNA LNPs significantly ameliorated chronic alcoholic liver injury in mice, and early treatment achieved the most profound effects. These effects can be attributed to a reduction in oxidative stress and increased expression of antioxidant genes, including *Gsh-Px*, *Gsh-Rd*, and *Sod1*. Moreover, we observed the alleviation of inflammation, evidenced by the downregulation of *Il-1β*, *Il-6*, *Tnf-α*, and *Tgf-β*, and the prevention of excessive lipid synthesis, evidenced by the restoration of the expression of *Srebp1c*, *Acc*, and *Fas*. Finally, triple siRNA treatment maintained normal metabolism in lipid oxidation. In brief, our research examined the possible targets for clinical intervention in ALD by examining the therapeutic effects of triple siRNA LNPs targeting *Cyp2e1*, *Cyp4a10*, and *Cyp4a14*. The in vivo knockdown of the three genes in this study is suggested as a promising siRNA therapeutic approach for ALD.

## 1. Introduction

In recent years, the incidence of disorders induced by excessive drinking has increased [1,2]. Long-term drinking may cause many diseases, and the alcohol-attributable fraction is the highest for liver cirrhosis [3]. The prevalence of alcoholic liver disease (ALD) is 4.8% worldwide [4]. Alcoholic liver diseases are liver conditions brought on by heavy drinking over an extended period. In the early stages, these typically manifest as fatty liver, which can progress to cirrhosis, liver fibrosis, and alcoholic hepatitis [5,6]. After long-term drinking, ethanol is metabolized in the liver, directly stimulating and damaging hepatocytes, thus affecting the normal metabolism and detoxification of proteins and fats in the liver. This, in turn, leads to alcoholic liver injury [7,8]. CYP2E1 is considered to be the enzyme most related to ALD because of its high catalytic activity for ethanol [9]. Long-term heavy drinking can increase the expression levels of CYP2E1 [10,11,12] and related drug transporters (such as Pgp) in the human body, but these can be restored to the non-induced state after several weeks of no ethanol exposure [13].

At present, drug therapy is the main means to treat alcoholic liver damage. Chemical inhibition of CYP2E1 attenuates alcohol-induced liver injury [14]. Clinical data have shown that clomethiazole improves human alcoholic liver disease by inhibiting CYP2E1 [15,16]. However, these traditional treatment drugs can only relieve symptoms and may have adverse effects on the gastrointestinal tract [17]. Fortunately, targeted RNA-interference (RNAi) therapies can promote the downregulation of specific genes [18,19] and have been developed as a promising therapeutic strategy for treating ALD. The knockout of CYP2E1 can alleviate alcohol-induced liver injury [20]. Our previous research showed that delivering si-*Cyp2e1* using lipid nanoparticles (LNPs) ameliorates ethanol-induced oxidative stress and ALD in vivo [21]. However, we subsequently found that some compensatory mechanisms could occur within CYP enzymes since si-*Cyp2e1* LNPs were not sufficient to inhibit the entire oxidative stress, and cytochrome P450-hydroxylase 4A (CYP4A) was increased. Analogously, CYP4A10 and CYP4A14 were compensatorily induced in *Cyp2e1*-null mice, with an increase in ROS production and oxidative stress [22]. This implies that CYP2E1 may not be the only CYP enzyme promoting oxidative stress. CYP4A10 and CYP4A14, as two key enzymes for *ω*-oxidation of hepatic fatty acids, should be considered as targets to be knocked down, along with CYP2E1 as a target to treat ALD. 

This study aimed to investigate the effects of triple siRNA LNPs simultaneously targeting *Cyp2e1*, *Cyp4a10,* and *Cyp4a14* on ameliorating alcohol-induced oxidative stress and liver injury.

## 2. Materials and Methods

### 2.1. Preparation and Characterization of Triple siRNA LNPs

siRNAs targeting mouse *Cyp2e1*, *Cyp4a10,* and *Cyp4a14* were designed as previously described [23]. The control sequence was previously described by Akinc et al. [24]. Single-stranded RNA was obtained from GenScript (Nanjing, China). The siRNA contained a 2′-*O*-methyl modification and dTsdT 3′ overhangs (where “s” denotes a phosphorothioate linkage) to improve its stability in vivo and inhibit immune stimulation. The LNPs were prepared using a microfluidic method [25]. The oil-phase components were Dlin-MC3-DMA, distearoyl phosphatidylcholine (Avanti Polar Lipids, Shanghai, China), cholesterol (Aladdin, Shanghai, China), and 1,2-dimyristoyl-sn-glycero-3-phosphoethanolamine-*N*-[methoxy(polyethylene glycol)-2000] (C14-PEG2000; Sigma-Aldrich, St Louis, MO, USA). These were dissolved in anhydrous ethanol at a molar ratio of 50/10/38.5/1.5 (*v*/*v*/*v*/*v*) and mixed with siRNA to allow for spontaneous particle formation [26]. The siRNAs were dissolved in 10 mM sodium acetate buffer (Coolaber, Beijing, China). The oil-phase liquid and aqueous-phase solution containing siRNA were mixed in a ratio of 1:3 using a microfluidic device (Precision Nanosystems, Vancouver, BC, Canada) at a rate of 12 mL/min. Samples were collected and dialyzed with a 20 K MWCO dialysis bag (Spectrum Laboratories, Rancho Dominguez, CA, USA) for 2 h at 25 °C using Dulbecco’s phosphate-buffered saline (DPBS; pH = 7.4) to remove the anhydrous ethanol. 

Triple siRNA LNPs were prepared [25] and characterized. The particle size, polydispersity index (PDI), and zeta potential of the LNPs were measured using a NanoBrook 90Plus PALS system (Brookhaven Instruments, Holtsville, NY, USA). The morphology of the LNPs was characterized using transmission electron microscopy (TEM) (Hitachi, Tokyo, Japan). The encapsulation rate was determined by using the Quant-iT™ RiboGreen^®^ RNA Assay [23] (Invitrogen, Grand Island, NY, USA).

### 2.2. Animal Experiments

Female C57BL/6N mice (6–8 weeks old) were purchased from Changzhou Cavins Laboratory Animal Co., Ltd.(Changzhou, China), and all mice were maintained under standard conditions (temperature, 22 ± 2 °C; relative humidity, 55 ± 5%; 12 h light/dark cycles).

In this study, si-*Cyp2e1*, si-*Cyp4a10*, and si-*Cyp4a14* were obtained after knocking down *Cyp2e1*, *Cyp4a10*, and *Cyp4a14* by RNAi. To verify the knockdown effect of si-*Cyp2e1*, si-*Cyp4a10*, and si-*Cyp4a14* in mice, the mice were divided into six groups (four mice/group). The control group was injected with DPBS only, while the remaining five groups were injected with triple siRNA LNPs. The expression of CYP2E1, CYP4A10, and CYP4A14 in the mice was detected on Days 1, 3, 5, 7, and 10 after injection. 

The chronic alcohol-fed mouse model was modified based on previous reports [27,28,29] to effectively mimic the characteristics of the different stages of human ALD and assess the effect of triple siRNA LNPs on the development of ALD. Specifically, eight-week-old female C57BL/6N mice were randomly divided into a control group, an alcohol-fed group, and nine dosing groups, each comprising eight mice, with a total of 88 mice. To ensure that every group of mice took the same amount of energy, each group was fed an equal caloric transition for a week. To enable the mice to progressively acclimate to alcohol irritation, the administration groups and the alcohol-fed group of mice were given a Lieber–DeCarli liquid diet (Dyets Biotechnology, Wuxi, China) [30] at progressively higher alcohol concentrations until the alcohol content reached 4%. Subsequently, the mice were given an ad libitum diet of a Lieber–DeCarli liquid containing 4% ethanol for a duration of 12 weeks, twice a week (5 g/kg, b.w.). The non-alcoholic liquid diet given to animals that were fed in pairs was calorie-matched.

**Figure 1:**
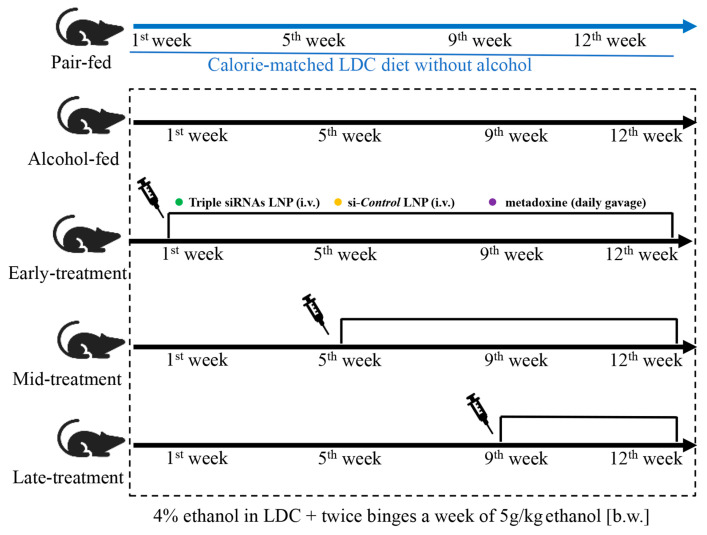
Diagram showing the administration of drugs for chronic alcoholic liver injury. LDC: Lieber–DeCarli; LNP: Lipid nanoparticle.

Triple siRNA LNPs and si-Control LNPs were administered via tail vein injection at a dose of 0.5 mg/kg. As a positive control, metadoxine (Shandong Qidu Pharmaceutical Co., Ltd., Shandong, China) [31] was administered intragastrically at a dose of 150 mg/kg/day. The specific administration method is shown in Figure 1 and Table 1. After the experiment, the mice were anesthetized, and blood was taken. The mice were sacrificed, and the fresh mouse liver was dissected. The whole blood of the mice was taken and placed at room temperature for 30 min and centrifuged (3000 rpm/min) for 20 min, and the upper serum was frozen. One-quarter of the fresh liver was taken in a 10% formalin solution, and paraffin-embedded sections were sectioned to observe the staining results of mouse sections. One-quarter of the liver of each mouse was taken for oil red O staining, and the remaining tissues were stored at −80 °C for protein and RNA preparation. No mice died in all groups during the experiment. From the third week of modeling, the mice in the model group began to drink and eat less, entering a state of burnout and weight loss; one week after treatment, the mice in the middle and late administration groups began to gain weight, increase their food and water intake, and show increased activity.

### 2.3. RNA Analysis

The livers of mice were dissected, and total RNA was extracted from the liver tissues using TRIzol reagent (Thermo Fisher Scientific, Waltham, MA, USA) according to the manufacturer’s instructions. The RNA concentration was measured using a NanoDrop™ 1000 spectrophotometer (Thermo Fisher Scientific, Waltham, MA, USA). cDNA was synthesized by reverse transcription according to the instructions of the BeyoRT™ cDNA First Strand Synthesis Kit (Beyotime Institute of Biotechnology, Shanghai, China). qRT-PCR experiments were performed using BeyoFast™ SYBR Green qPCR Mix and a Light Cycle 96 Real-Time PCR system (Roche, Basel, Switzerland). The qRT-PCR program comprised a reverse transcription step at 48 °C for 30 min and a Taq polymerase activation step at 95 °C for 300 s. This was followed by PCR: 45 cycles at 95 °C for 15 s, 61 °C for 20 s, and 72 °C for 30 s, followed by a final melting cycle. The 2^−ΔΔCT^ method was used to analyze the data, with *Gapdh* as the reference gene, following the instructions for the fluorescence quantification kit [32]. Primers for the relevant target genes are listed in Table S2. 

### 2.4. Protein Analysis

Liver microsomes were extracted from the liver tissue using KCl sucrose buffer, and total protein from the mouse liver tissue was lysed in RIPA buffer (P0013B; Beyotime Biotechnology, Shanghai, China) according to the manufacturer’s instructions. A BCA protein concentration analysis kit (P0011, Beyotime Biotechnology, Shanghai, China) was used to quantify the protein concentrations of the partially extracted liver microsomes and lysates. Western blotting was used to detect the protein expression of CYP2E1 (BML-CR3271, Enzo Biochem, New York, NY, USA) and CYP4A (sc-271983, Santa Cruz Biotechnology, Santa Cruz, CA, USA) in the liver microsomes. The protein expression of DPBS (SREBP1c, PAC868Mu01, CLOUD-CLONE CORP, Wuhan, China), fatty acid synthase (FAS, PAC470Mu01, CLOUD-CLONE CORP, Wuhan, China), peroxisome proliferator-activated receptor-γ coactivator-1α (PGC-1α, PAH337Mu01, CLOUD-CLONE CORP, Wuhan, China), carnitine palmitoyl transferase 1 (CPT1, PAF368Mu01, CLOUD-CLONE CORP, Wuhan, China), and glutathione peroxidase 4 (GPX4, A1933, ABclonal, Wuhan, China) in total mouse liver protein was detected with Western blotting. The extracted proteins were subjected to sodium dodecyl sulfate-polyacrylamide gel electrophoresis (SDS-PAGE) in the protein loading buffer at a ratio of 5:1 to solubilize the liver microsomes and total liver proteins. The solution was placed in a boiling water bath for 5 min to denature and inactivate the proteins and then stored at −20 °C. Equal amounts of protein were loaded onto 10–12% SDS-PAGE gels and then transferred to polyvinylidene fluoride (PVDF, Millipore, Billerica, MA, USA) membranes. The primary antibody solution was diluted at a ratio of 1:1000, cultured overnight at 4 °C, and then incubated with HRP-coupled secondary antibody (dilution ratio 1:3000) (Beyotime Biotechnology, Shanghai, China). Protein bands were detected using an enhanced chemiluminescence reagent. Chemiluminescent signals were visualized and analyzed using the ChemiDoc XRS imaging system (Bio-Rad, Hercules, CA, USA). Calnexin (10427-2-AP; Proteintech Group, Rosemont, IL, USA) was used as a loading control for microsomal samples, and GAPDH (ab9485; Abcam, Cambridge, MA, USA) was used as a loading control for total mouse liver protein. 

### 2.5. Measurement of Mouse Liver Index and Biochemical Assays

The livers of each group of mice were washed with 0.9% ice-cold saline, and any water remaining on the surface was blotted. The liver was observed and weighed to calculate the liver index (liver weight/body weight). Blood was collected, and serum was prepared by centrifugation (3500 rpm, 10 min). Serum alanine aminotransferase (ALT) and aspartate transaminase (AST) levels were measured using assay kits. Liver tissue homogenate samples were prepared in ice-cold saline (1:9, *w*/*w*) at 2500 rpm and centrifuged for 10 min, and the liver catalase (CAT), ROS, GSH, glutathione peroxidase (GSH-PX), malondialdehyde (MDA), superoxide dismutase (SOD), triglyceride (TG), and total cholesterol (TC) concentrations and the iron content were measured using the respective assay kits. All of the above commercial kits were purchased from Nanjing Jiancheng Bioengineering Institute (Nanjing, China) and performed according to the manufacturer’s instructions. The ROS levels in each group were normalized to the ratio in the pair-fed group.

### 2.6. Liver Histomorphology

Two pieces of liver tissue with an approximate size of 5 mm were cut from the liver of each mouse. One piece of liver tissue was fixed in 10% neutral formalin buffer, followed by paraffin embedding and sectioning (5 μm thick), and the cut paraffin sections were stained with hematoxylin–eosin, Sirius red, and fluorescent immunomarkers. Macrophage infiltration was analyzed using fluorescent immunohistochemical staining with F4/80 (GB113373, Servicebio; Wuhan, China), and at least six randomly selected 200 × fields of view were photographed for each section within each group. Photographs were taken such that as much tissue as possible filled the entire field of view, ensuring that the background light was consistent for each photograph. The other piece of fresh liver tissue was embedded with an embedding agent and frozen at −80 °C. Sections (10 μm) were stained with Oil Red O. Frozen sections were thawed to room temperature and observed for Oil Red O staining and liver lipid accumulation using an inverted microscope (Nikon, Tokyo, Japan). Optical microscope photographs were taken, and the number of hepatocyte fat droplets in the frozen sections was measured using the Image-Pro Plus 6.0 (Media Cybernetics, Rockville, MD, USA) image analysis software.

### 2.7. Statistical Analysis

All experimental data were calculated using the mean ± standard deviation and one-way analysis of variance (ANOVA) using SPSS 20.0 software (IBM, Armonk, NY, USA) for comparison between groups, and *p* < 0.05 was considered to indicate statistical significance.

## 3. Results

### 3.1. Characterization and Knockdown Effects of Triple siRNA LNPs In Vivo

**Figure 2:**
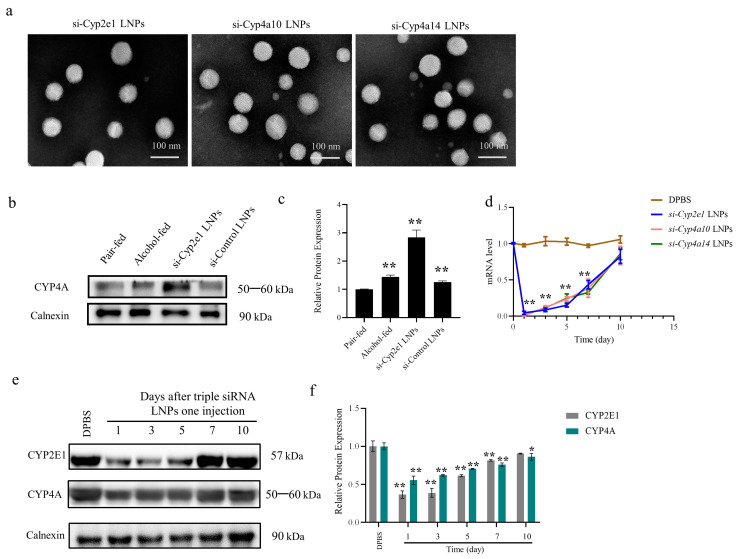
Knockdown effects of triple siRNA LNPs in vivo. (**a**) Representative transmission electron microscopy images of LNPs encapsulated with triple siRNA. (**b**,**c**) Effect of knocking down *Cyp2e1* gene in an alcohol mouse model on CYP4A. (*n* = 4). ** *p* < 0.01 versus pair-fed. (**d**) Changes over time in *Cyp2e1*, *Cyp4a10*, and *Cyp4a14* mRNA levels in mice injected with a single dose of triple siRNA LNPs. ** *p* < 0.01 versus pair-fed. (**e**,**f**) Changes in CYP2E1 and CYP4A protein levels over time in mice injected with a single dose of triple siRNA LNPs. Data are expressed as mean ± standard deviation (SD) (*n* = 8). * *p* < 0.05 and ** *p* < 0.01 compared with Dulbecco’s phosphate-buffered saline (DPBS) as control; n.s. means not significant. LNPs: lipid nanoparticles.

The size distribution, PDI, and siRNA encapsulation rates of the si-*Cyp2el*, si-*Cyp4a10*, and si-*Cyp4a14* LNPs are shown in Table 2. TEM images showed that the morphologies of si-*Cyp2el*, si-*Cyp4a10*, and si-*Cyp4a14* LNPs were spherical, and the particle size range was further determined to be consistent with the results of the particle size analyzer (Figure 2a).

The knockdown of CYP2E1 by si-*Cyp2e1* LNPs increased CYP4A protein expression in vivo (*p* < 0.01, Figure 2b,c), demonstrating that CYP4A can act as an alternative initiator of oxidative stress when the *Cyp2e1* is downregulated.

The expression levels of *Cyp2e1*, *Cyp4a10*, and *Cyp4a14* were assessed in mouse livers at different time points after a single injection. Real-time PCR analysis confirmed that on day 1 after LNP-siRNA injection, the mRNA expression of the three genes was reduced by approximately 90% compared with that in the normal group. As the gene knockdown of RNAi is recoverable in vivo, the resultant graph shows that the normal state could be restored after 10 days (*p* < 0.01, Figure 2d). Western blotting results showed that CYP2E1 and CYP4A protein levels decreased to 30% one day after injection and remained at 80% on day 7 after injection (*p* < 0.05, Figure 2e,f). Based on the silencing effect of the triple siRNA LNPs in mice, ALD treatment was performed using a weekly dosing regimen.

### 3.2. Effects of Triple siRNA LNPs on Chronic Alcohol-Induced Liver Injury

**Figure 3:**
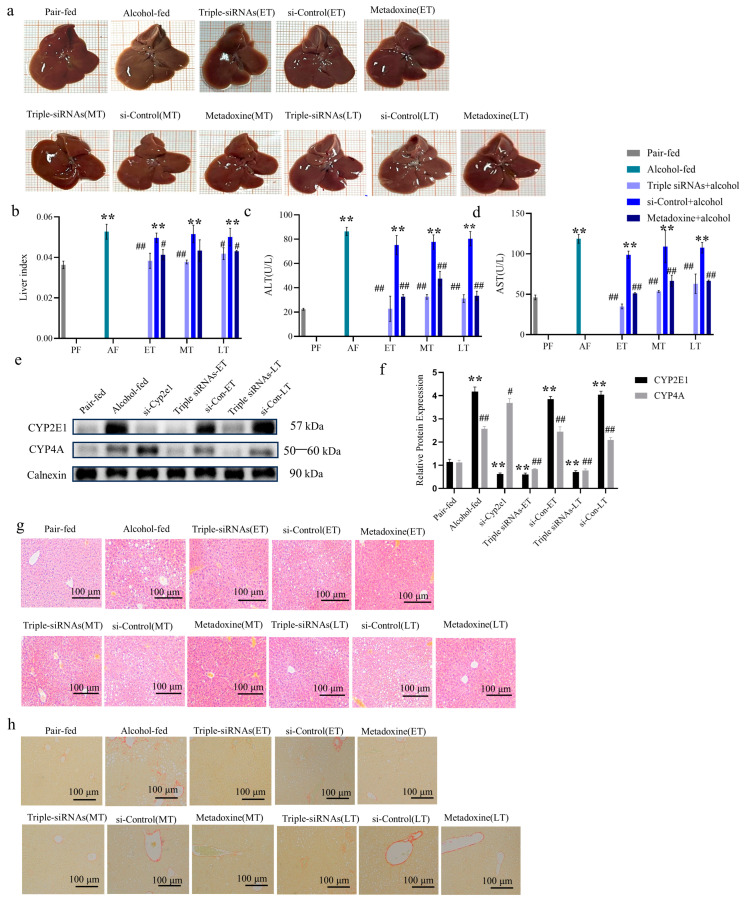
The role of triple gene knockdown in the treatment of alcoholic liver injury. (**a**) Gross morphology of the liver of mice in each group. (**b**) Effect of triple siRNA LNP administration on the liver index. (**c**,**d**) Effect of triple siRNA LNPs on serum AST and ALT levels in mice with alcoholic liver injury. (**e**,**f**) The protein expression levels of CYP2E1 and CYP4A. (**g**) Histopathological sections of the mouse liver were stained with hematoxylin and eosin (200×). (**h**) Sirius scarlet staining of the histopathological sections of mouse liver (200×). Data are expressed as the mean ± SD (*n* = 8). ** *p* < 0.01 versus pair-fed; # *p* < 0.05 and ## *p* < 0.01 versus alcohol-fed. PF/AF/ET/MT/LT: Pair-fed/Alcohol-fed/Early-treatment/Mid-treatment/Late-treatment.

The liver shape and index of each group of mice were examined at different stages of ALD administration of triple siRNA LNPs. The results of these effects are shown in Figure 3. The model group had dark-colored livers with a slightly tougher and rougher surface texture, and a distinctly granular appearance of the liver could be observed with the naked eye. There was no significant difference between the early triple siRNA LNP administration group and the control group, which had appropriately sized, red-colored, soft livers. The positive control group, which received metadoxine, showed good improvement compared with the liver of the alcoholic model group, but there were certain phenotypes of alcohol infiltration damage to the liver (Figure 3a). The liver index of mice in the alcohol-fed group was significantly higher than that of the control group (*p* < 0.01, Figure 3b), whereas the liver index of the mice that were administered triple siRNA LNPs was significantly lower than that of the alcohol-fed group (*p* < 0.05, Figure 3b). This suggests that chronic alcohol consumption induces an increase in the liver index, and triple siRNA LNPs can mitigate this damage.

Biochemical analyses of serum samples from each group of mice were performed. Compared with the control group, the levels of AST and ALT in the alcohol-fed group were significantly increased. These were then remarkably decreased after triple siRNA LNP administrations (*p* < 0.01, Figure 3c,d), indicating that the knockdown of *Cyp2e1*, *Cyp4a10,* and *Cyp4a14* could inhibit the alcohol-induced increase in AST and ALT levels in mice. Moreover, the early treatment group had lower mean AST and ALT levels than the middle and late treatment groups, indicating that early intervention is beneficial to ALD recovery (Figure 3c,d).

Mouse liver microsome samples were prepared at the end of animal treatment. After 12 weeks of alcohol feeding, the expression of the CYP2E1 protein increased by about 4.17 times in the alcohol-fed group compared with the pair-fed group. After 12 weeks of continuous tail vein injection of triple siRNA LNPs, compared with the si-Control group, the expression of the CYP2E1 protein in the early-treatment group decreased by >80%. In addition, 12 weeks after injection of si-*Cyp2e1* LNPs, CYP2E1 knockdown was accompanied by an increase in CYP4A protein levels. After injection of triple siRNA LNPs, CYP4A protein expression decreased by >60% in the early treatment group (*p* < 0.01, Figure 3e,f).

Microscopic observation of the liver sections stained with hematoxylin–eosin in each group showed that the hepatocytes in the control group had a neat and uniform structure with clearly visible nuclei, while the hepatocytes in the mice in the alcohol model group and the si-Control group at different stages had a light cytoplasmic color, disorganized cell arrangement, gradually increasing inflammatory factor infiltration, and obvious fatty degenerative vacuoles. In contrast, the hepatocytes of the mice that were administered triple siRNA LNPs were not affected by alcohol and were neatly arranged; the livers of the metadoxine group showed less vacuolation, despite the treatment effect (Figure 3g). Therefore, the administration of triple siRNA LNPs significantly reduced chronic alcohol-induced damage to the liver.

The results of the Sirius scarlet staining indicated that both the alcohol-fed group and the different stages of the si-Control LNPs group showed significant scarlet collagen fibrillation compared with the control group, whereas fibrosis was significantly reduced after treatment with triple siRNA LNPs; a small amount of fibrosis was still present in the positive control despite the treatment effect (Figure 3h). This indicates that the triple siRNA LNPs had a significant protective effect against alcohol-induced liver injury, with the most obvious protective effect observed in the early treatment group.

### 3.3. Effects of Triple siRNA LNPs on the Results of Oxidative Stress and Lipid Correlation in ALD

**Figure 4:**
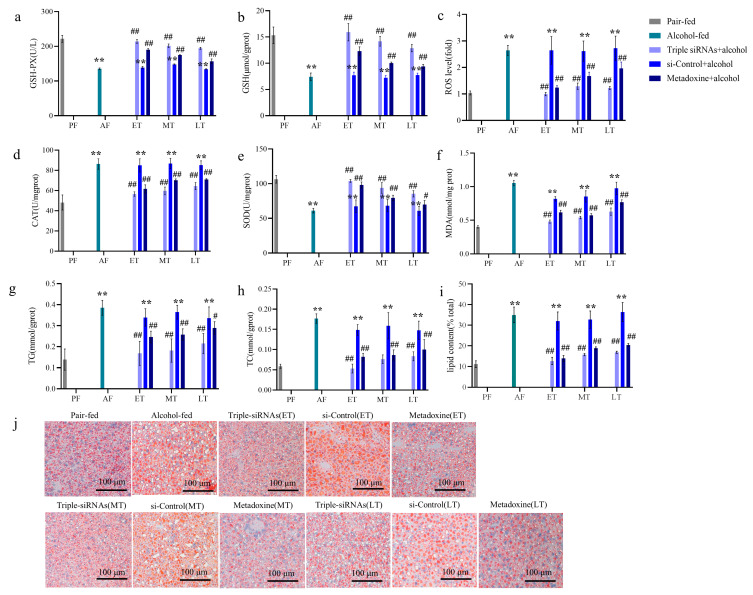
Antioxidant levels in liver tissue and the effect of triple siRNA LNPs on blood lipid levels in mice. (**a**–**f**) Effect of si-*Cyp2e1* LNP on hepatic glutathione peroxidase (GSH-PX), glutathione (GSH), reactive oxygen species (ROS), catalase (CAT), superoxide dismutase (SOD), and malondialdehyde (MDA) levels in alcoholic liver-injured mice. (**g**,**h**) Effect of triple siRNA LNPs on hepatic triglyceride (TG) and total cholesterol (TC) levels in mice with alcoholic liver injury. (**i**) Quantification of Oil Red O staining in different groups. (**j**) Histopathological sections of the mouse liver stained with Oil Red O (200×). Orange-red represents obvious fat accumulation in the liver. Data are expressed as the mean ± SD (*n* = 8). ** *p* < 0.01 versus pair-fed; # *p* < 0.05 and ## *p* < 0.01 versus alcohol-fed. PF/AF/ET/MT/LT: Pair-fed/Alcohol-fed/Early-treatment/Mid-treatment/Late-treatment.

Compared with the control group, the levels of GSH-Px and GSH in the alcohol-fed group were significantly reduced, as observed in the findings of oxidative-stress-related indicators (*p* < 0.01, Figure 4a,b). The administration of triple siRNA LNPs restored GSH depletion (*p* < 0.01, Figure 4b). An analysis of the ROS levels in fresh liver tissues showed that the ROS levels were significantly higher in the alcohol-fed group than in the control group (*p* < 0.01, Figure 4c). Moreover, the antioxidant indicator SOD in the alcohol-fed group was significantly decreased (*p* < 0.01, Figure 4e), whereas hepatic CAT and MDA were elevated (*p* < 0.01, Figure 4d,f), suggesting an increase in oxidative stress secondary to the lipid peroxidation process. In contrast, the administration of triple siRNA LNPs significantly reversed these changes. This suggests that alcohol metabolism generates free radicals in the liver and that chronic alcohol consumption leads to elevated levels of oxidative stress in the body. Triple siRNA LNPs can reduce oxidative stress by restoring the antioxidant enzymes and GSH levels, making early prevention more effective.

Changes in hepatic TG and TC levels were analyzed in each group of mice. Liver TG and TC levels in alcohol-fed mice were significantly higher than those in the pair-fed group, indicating that excessive alcohol consumption disrupted the normal metabolism of lipids and induced a disturbance in hepatic lipid metabolism. After treatment with triple siRNA LNPs, the liver TG and TC levels were significantly decreased (*p* < 0.01, Figure 4g,h), with better results in the early treatment group.

Further histological assessment by means of Oil Red O staining (Figure 4j) showed that prolonged alcohol feeding led to lipid droplet accumulation in the livers of mice, whereas the administration of triple siRNA LNPs significantly reduced the orange-red lipid droplet accumulation in the livers. Analyses of the lipid droplets showed that the lipid accumulation in the liver of mice fed with alcohol was significantly increased compared with the control group, while it was decreased after triple siRNA LNPs treatments (*p* < 0.01, Figure 4i).

### 3.4. Effects of Triple siRNA LNPs on the Inflammation Level of ALD

**Figure 5:**
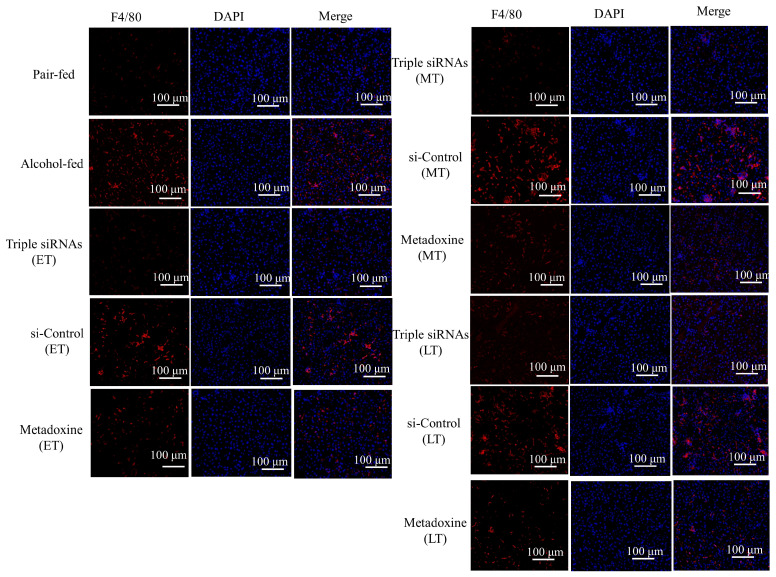
Fluorescence immunohistochemistry of F4/80 (red) in the mouse liver; the nuclei are stained with DAPI (blue) (200×). ET/MT/LT: Early-treatment/Mid-treatment/Late-treatment.

The presence of macrophages was positively correlated with positive F4/80 expression. Normal livers showed low positive expression of F4/80 in the fluorescence immunohistochemistry results, with light red fluorescence present. A significant increase in the F4/80 red fluorescence expression was observed in mice in the alcohol model group and the si-Control LNPs group at all stages. No obvious red fluorescence expression was observed in the liver tissue of mice in the triple siRNA LNP administration group, and the triple siRNA-ET had an especially significant protective effect on the liver, with the expression being approximately the same as in the control group. The mice who were given metadoxine in the three different periods also showed a decrease in F4/80 fluorescence expression, with the best treatment effects in the early stage. However, triple siRNA LNP administration showed a more pronounced reduction in the positive F4/80 expression than that of metadoxine (Figure 5). The above fluorescence results showed that the administration of triple siRNA LNPs protected the liver from alcohol, especially after early administration, and prevented the inflammatory response. 

### 3.5. Effects of Triple siRNA LNPs on the Expression of Genes Related to Inflammatory Factors, Oxidative Stress, and Steatosis

**Figure 6:**
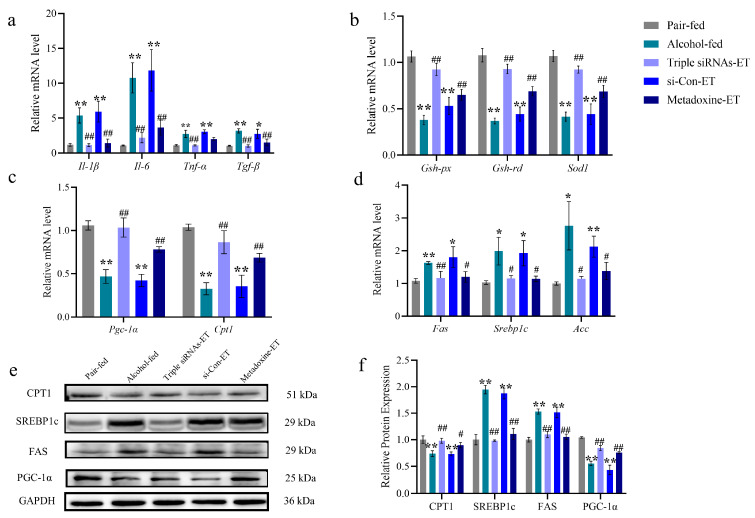
Role of triple siRNA LNPs in oxidative stress and levels of lipid- and inflammation-related genes and proteins. (**a**) mRNA expression levels of *Il-1β*, *Il-6*, *Tnf-α*, and *Tgf-β*. (**b**) mRNA expression levels of *Gsh-px*, *Gsh-rd*, and *Sod1*. (**c**) The mRNA expression levels of *Cpt1* and *Pgc-1α*. (**d**) The mRNA expression levels of *Fas*, *Srebp1c*, and *Acc*. (**e**,**f**) Relative protein expression levels of CPT1, SREBP1c, FAS, and PGC-1α. Data are expressed as mean ± SD (*n* = 8). * *p* < 0.05 and ** *p* < 0.01 versus pair-fed; # *p* < 0.05 and ## *p* < 0.01 versus alcohol-fed. The mRNA levels are relative to the levels of the reference gene *Gapdh*, and the protein levels are relative to the levels of the reference protein GAPDH. ET: Early-treatment.

Total mouse mRNAs and proteins were prepared from the livers of mice in the pair-feeding, alcohol-feeding, triple siRNA-ET, si-Con-ET, and metadoxine-ET groups at the end of the treatments. Regarding the expression of mRNAs related to the relevant inflammatory factors, alcohol-induced expression of *Il-1β*, *Il-6*, *Tnf-α*, and *Tgf-β*, and triple siRNA LNP administrations reduced these raised levels (*p* < 0.01, Figure 6a). Triple siRNA LNPs restored the decreased expression of the oxidative-stress-related factors *Gsh-px*, *Gsh-rd,* and *Sod1* to normal levels (*p* < 0.01, Figure 6b). Triple siRNA LNPs enhanced the *β*-oxidation of fatty acids by increasing the mRNA expression of *Cpt1*. Alcohol reduced the expression level of *Pgc-1α*, altering the intracellular redox homeostasis; in contrast, triple siRNA LNPs increased the expression of *Pgc-1α* in vivo (*p* < 0.01, Figure 6c). Metabolic pathways that regulate lipid synthesis and fatty acid oxidation in vivo can control fatty acid metabolism and lipid retention in the liver. The expression of genes responsible for regulating lipid metabolism in the liver was analyzed, and results showed that alcohol feeding upregulated the expression of *Srebp1c*, *Acc*, and *Fas*, which are genes related to fatty acid synthesis (*p* < 0.05, Figure 6d). Consistent with mRNA expression, the protein levels of SREBP-1c and FAS were significantly reduced and the protein levels of CPT1 and PGC-1α were significantly upregulated after the administration of triple siRNA LNPs, compared with those in the alcohol-fed group (*p* < 0.01, Figure 6e,f).

### 3.6. Effects of Triple siRNA LNPs on Ferroptosis in Hepatocytes

**Figure 7:**
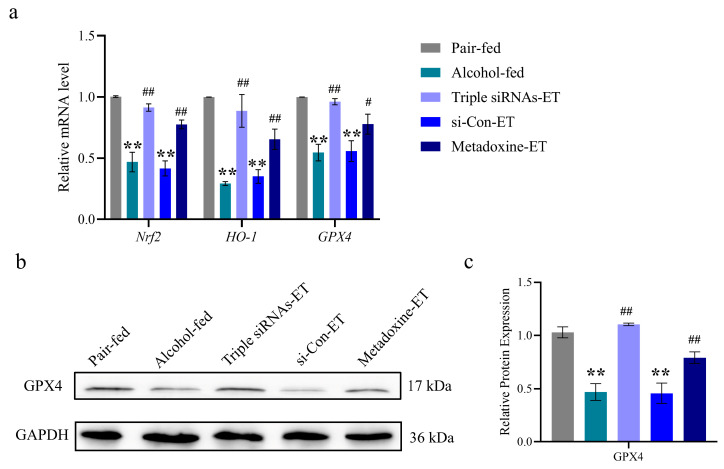
Effects of triple siRNA LNPs on alcohol-induced ferroptosis in hepatocytes. (**a**) The mRNA expression levels of *Nrf2*, *HO-1*, and *GPX4*. (**b**,**c**) The relative protein expression levels of GPX4. Data are expressed as mean ± SD (*n* = 8). ** *p* < 0.01 versus pair-fed; # *p* < 0.05 and ## *p* < 0.01 versus alcohol-fed. The mRNA levels are relative to the levels of the reference gene *Gapdh*, and the protein levels are relative to the levels of the reference protein GAPDH. ET: Early-treatment.

The iron content of the liver tissue was significantly increased in the alcohol-fed group. The administration of the triple siRNA LNPs interfered with the accumulation of iron in hepatocytes and maintained their normal state (Table 3). In addition, we detected the expression levels of ferroptosis-related factors. The mRNA expression levels of *Nrf2* and *HO-1* were significantly lower in the alcohol-fed and si-Control groups than in the control group. There was no significant difference in the expression of relevant genes between the triple siRNA LNPs group and the control group (*p* < 0.01, Figure 7a). Simultaneously, the expression of GPX4, the core regulatory protein of ferroptosis, was analyzed quantitatively. Compared with the control group, the mRNA and protein expression levels of GPX4 in the alcohol gavage group were significantly decreased (*p* < 0.01, Figure 7b,c), indicating that the occurrence of iron death is involved in alcohol-induced liver injury.

## 4. Discussion

ALD is a global health concern that is widely believed to be related to oxidative stress formed by ROS [33]. CYP2E1 is one of the major ROS generators. It acts by catalyzing microsomal ethanol metabolism in the liver and is considered to contribute to ALD. Ethanol is metabolized by alcohol dehydrogenase and microsomal cytochrome P450 (CYP) enzymes to produce acetaldehyde and reactive oxygen species (ROS), which consume glutathione (GSH) [34,35,36]. The production of ROS is crucial for the maintenance of steatosis and steatohepatitis throughout the progression of ALD and is additionally related to liver inflammation [33]. ROS from damaged cells activate inflammatory immune cells and further enhance oxidative stress by producing ROS and active nitrogen species [37]. Hepatocytes exposed to alcohol undergo oxidative-stress-induced cell damage and death, thus producing various inflammatory mediators, such as cytokines and chemokines, which in turn activate immune responses and inflammation [38,39,40,41]. Additionally, iron overload in the liver can enhance oxidative stress through the Fenton reaction, promote the activation of Kupffer cells, and induce and aggravate alcohol-induced liver injury [42]. These findings highlight the importance of oxidative stress in the pathogenesis of ALD.

Although increasing evidence has indicated the role of ethanol-inducible CYP2E1 in oxidative stress, *Cyp2e1*-null mice still develop steatohepatitis with a dramatic increase in CYP4A gene expression [43]. Similarly, this study also found that si-*Cyp2e1*-downregulated CYP2E1 expression was accompanied by a markedly elevated CYP4A protein level in vivo (Figure 2a,b). Studies have shown that CYP450 of the 4A family is the driving force for liver injury in β-thalassemia mouse models [44]. These studies strongly suggest that CYP4A may also play an important role in the progression of ALD. The CYP4 family includes 11 subfamilies (CYP4A-CYP4M), which encode constitutive and inducible isoenzymes, respectively [45]. *CYP4A10* and *CYP4A14* in mice (homologous to human *CYP4A22* and *CYP4A11*, respectively) are highly expressed in the liver [46] and are mainly responsible for the *ω*-oxidation of hepatic fatty acids, which is an effective source of ROS through the uncoupling of the catalytic cycle, thus stimulating oxidative stress pathways [43]. In this study, triple siRNA LNPs were used to inhibit the expression of *Cyp2e1*, *Cyp4a10*, and *Cyp4a14* in mice. The pathogenesis of ALD is related to oxidative stress, lipid metabolism disorder, ferroptosis, and inflammatory responses. We found that early intervention of triple siRNA LNPs can significantly improve the symptoms of ALD, thus successfully reducing its occurrence.

RNAi is an efficient and specific degradation of highly conserved homologous mRNAs during evolution and regulates the expression of targeted genes in diseases with complex mechanisms in vivo to achieve therapeutic effects [47]. Owing to the large size, strong hydrophilicity, and anionic charge of siRNA molecules, their cellular delivery can be challenging; therefore, appropriate drug-delivery carriers are needed [48]. LNPs have evolved into a promising delivery vehicle with much fewer immunogenic and toxic effects than viral vectors [49]. siRNA LNPs can specifically suppress one or more genes at the same time, and multiplex siRNA-targeted regulation makes personalized medicine possible [50]. The triple siRNA LNPs we developed enabled the simultaneous regulation of multiple CYPs in vivo, suggesting the possibility of achieving precise metabolic regulation in vivo in the future. 

Recent advances demonstrated that si-*Cyp2e1* partly decreased ROS generation and oxidative stress, liver fat accumulation, hepatocyte inflammation, and fibrosis in mouse livers, contributing to improved ALD symptoms in mice [21]. However, the research was limited to a single endpoint, ignoring the compensatory mechanism of CYP4A in oxidative stress pathways. The current study utilized triple siRNA LNPs to achieve RNAi-mediated multiplex knockdown of *Cyp2e1*, *Cyp4a10*, and *Cyp4a14* simultaneously, which ameliorated the chronic alcoholic liver injury in mice by reducing oxidative stress and improving antioxidant capacity, alleviating inflammation, preventing excessive lipid synthesis, maintaining normal lipid oxidation metabolism, and suppressing ferroptosis. The triple siRNA LNPs had both preventive and therapeutic effects in mice with chronic alcoholic liver injury and were more effective than metadoxine, the positive drug used in this study. Moreover, the early treatment group achieved the best preventive effect through three-stage treatments of ALD, indicating that the earlier the administration, the more obvious the efficiency.

Excessive iron accumulation can induce liver injury by promoting oxidative stress and iron-dependent cell death, that is, deironized anemia [51,52]. Mice that were fed alcohol demonstrated signs of iron overload in the liver, as well as reduced GPX4 activity, a biomarker of low-iron anemia [53]. NRF2 is a key negative regulator of ferroptosis and can play a strong antioxidant/apoptotic role [54]. Heme oxygenase 1 (HO-1) can regulate oxidative stress indicators, thereby reducing cell ferroptosis-induced damage and restoring normal cell function [55]. In our study, triple siRNA LNPs increased the mRNA expression of *Nrf2*, *HO-1*, and *GPX-4*, thereby reducing the production of ROS and making cells less susceptible to sensitization. Our findings revealed that triple siRNA LNPs, which regulate ferroptosis, served as a strategy to decrease and repair damage induced by oxidative stress, presenting a novel target for disease treatment [21]. Reducing iron accumulation and lipid peroxidation and regulating the expression of ferroptosis-related genes are further methods for the treatment of ALD. 

Ethanol promoted lipid synthesis in vivo. PGC-1α mainly regulates oxidative metabolism in vivo [56], and CPT1 is the rate-limiting enzyme in fatty acid *β*-oxidation [57]. SREBP-1c, FAS, and ACC are involved in hepatic lipid synthesis [58]. Ethanol upregulated the mRNA expression of *Srebp1c*, *Acc*, and *Fas* and increased the synthesis of TG and TC, leading to lipid accumulation [59]. The CYP4A subfamily are fatty acid hydroxylases that catalyze the *ω*-hydroxylation of medium and long-chain fatty acids [60]. Numerous studies claim that an increase in rodent CYP4A occurs during hepatic steatosis and steatohepatitis [61,62,63], and CYP4A10 and CYP4A14 are pronouncedly induced owing to the increased accumulation of lipid peroxides [43]. As expected, triple siRNA LNPs targeting *Cyp2e1*, *Cyp4a10*, and *Cyp4a14* downregulated the mRNA expression of *Srebp1c*, *Acc*, and *Fas*. Therefore, the protective effect of the triple siRNA LNPs against ALD may be related to the regulation of lipid metabolism.

CYP4A10 and CYP4A14 are also responsible for omega-hydroxylation of arachidonic acid apart from fatty acids and regulate the host inflammatory response [64]. The absence of *Cyp4a10* and *Cyp4a14* has been shown to attenuate or abrogate the enteropathogenic bacterial infection [60]. The downregulation of CYP4A14 has been shown to have an anti-inflammatory response in the intestine, resulting in reduced oxidative stress and the amelioration of dextran-sulfate-sodium-induced colitis [65]. Consistent with these results, our findings revealed that triple siRNA LNPs inhibited macrophage activation and downregulated the expression of *Il-6*, *Tnf-a*, and *Tgf-β*, thereby reducing the inflammatory response in ALD.

Non-alcoholic steatohepatitis (NASH) is a common liver injury with similar pathological features to ALD [33]. The changes in gene regulation and expression of CYP may reflect the early stage of NASH and may be beneficial for the treatment of hepatic steatosis. A previous study showed that CYP2E1 plays a critical role in NASH development by promoting oxidative/nitrosative stress, protein modifications, inflammation, and insulin resistance [66]. Further research verified that CYP2E1 deficiency neither prevented the development of NASH nor eliminated microsomal lipid peroxidation, suggesting the presence of other compensatory pathways. In addition, CYP4A10 and CYP4A14 were upregulated in *Cyp2e1*-null NASH mice, and hepatic microsomal lipid peroxidation was essentially inhibited when implemented with anti-mouse CYP4A10 antibody in vitro [22]. Additionally, the ablation of the CYP4A14 gene markedly attenuated liver damage, inflammation, and fibrosis in NASH [67]. The above evidence could support our conjecture that CYP2E1, CYP4A10, and CYP4A14 may also be contributing factors to the pathogenesis of NASH. Further studies involving this multiplexed RNAi approach to NASH should be carried out. 

## 5. Conclusions

In conclusion, we successfully developed a multiplexed RNAi strategy for *Cyp2e1*, *Cyp4a10*, and *Cyp4a14* genes in mice, conceivably revealing the role of P450-related oxidative stress in ALD. This evidence could suggest potential targets and clinical intervention approaches for future ALD therapies. 

## Figures and Tables

**Table 1:** Schedule of treatment group.

Group	Therapeutic Time
Triple siRNA-ET	Early-treatment (1st week to 12th week)
si-Con-ET	
Metadoxine-ET	
Triple siRNA-MT	Mid-treatment (5th week to 12th week)
si-Con-MT	
Metadoxine-MT	
Triple siRNA-LT	Late-treatment (9th week to 12th week)
si-Con-LT	
Metadoxine-LT	

ET/MT/LT: Early-treatment/Mid-treatment/Late-treatment.

**Table 2:** Particle size distribution, polydispersity index (PDI), zeta potential, and encapsulation rate of lipid nanoparticles (LNPs).

Gene	Size (nm)	PDI	Zeta Potential (mV)	Encapsulation Rate (%)
*Cyp2e1*	75.34 ± 1.47	0.189 ± 0.003	−0.55 ± 0.1	91.9 ± 0.01
*Cyp4a10*	84.96 ± 3.92	0.198 ± 0.002	−0.67 ± 0.1	92.8 ± 0.01
*Cyp4a14*	85.36 ± 1.34	0.180 ± 0.019	−0.38 ± 0.1	92.2 ± 0.01

**Table 3:** Liver tissue iron contents.

Group	Liver Tissue Iron Content (μg/mg prot)
Pair-fed	2.12 ± 0.23
Alcohol-fed	3.80 ± 0.26 **
Triple siRNAs -ET	2.26 ± 0.19 ^##^
si-Con-ET	3.91 ± 0.09 **
Metadoxine-ET	2.93 ± 0.14 ^##^

** *p* < 0.01 versus pair-fed; ^##^ *p* < 0.01 versus alcohol-fed. ET: Early-treatment.

## Data Availability

Data are contained within the article and Supplementary Materials.

## Supplementary Materials

The following supporting information can be downloaded at https://www.mdpi.com/article/10.3390/biomedicines12071505/s1. Table S1: Sequences of small interfering RNAs (siRNAs) targeting *Cyp2e1*; Table S2: Primer sequences for qPCR.

## Abbreviations

ALD: Alcoholic liver disease; ALT: Alanine aminotransferase; AST: Aspartate transaminase; CAT: Catalase; CPT1: Carnitine palmitoyl transferase 1; CYP: Cytochrome P450; FAS: Fatty acid synthase; GPX4: Glutathione peroxidase 4; GSH: Glutathione; GSH-PX: Gluta-thione peroxidase; LNPs: Lipid nanoparticles; MDA: Malondialdehyde; PDI: Polydispersity index; PGC-1α: Peroxisome proliferator-activated receptor-γ coactivator-1α; RNAi: RNA-interference; ROS: Reactive oxygen species; SOD: Superoxide dismutase; SREBP-1c: Sterol regulatory element binding protein-1c; TEM: Transmission electron microscopy; TC: Total cholesterol; TG: Triglyc-eride.
