# Supplementary material for: Reducing Oxidative Stress-Mediated Alcoholic Liver Injury by Multiplexed RNAi of Cyp2e1, Cyp4a10, and Cyp4a14
Source: Biomedicines. 2024 Jul 6;12(7):1505. doi: 10.3390/biomedicines12071505 (PMC11274525; doi:10.3390/biomedicines12071505)
Supplement: Supplementary file 1 [file biomedicines-12-01505-s001.zip › Table S1.pdf]

Table S1 Sequences of small interfering RNAs (siRNAs) targeting *Cyp2e1*.

Gene	siRNA	Sequence
Cyp2e1	si-Cyp2e1 sense	ccAuGuAcAcAAuGGAAAAdTsdT
	si-Cyp2e1 antisense	UUUUCcAUUGUGuAcAUGGdTsdT
Cyp4a10	*si-* Cyp4a10 *sense*	ccuucuGAAuuAuGAGGAAdTsdT
	si- Cyp4a10 *antisense*	UUCCUcAuAAUUcAGAAGGdTsdT
Cyp4a14	si-Cyp4a14 sense	cuGucuGucucAuAGuuAAdTsdT
	si-Cyp4a14 antisense	UuAACuAUGAGAcAGAcAGdTsdT
Control	si-control sense	cuuAcGcuGAGuAcuucGAdTsdT
	si-control antisense	UCGAAGuACUcAGCGuAAGdTsdT
