# Supplementary material for: Reducing Oxidative Stress-Mediated Alcoholic Liver Injury by Multiplexed RNAi of Cyp2e1, Cyp4a10, and Cyp4a14
Source: Biomedicines. 2024 Jul 6;12(7):1505. doi: 10.3390/biomedicines12071505 (PMC11274525; doi:10.3390/biomedicines12071505)
Supplement: Supplementary file 1 [file biomedicines-12-01505-s001.zip › Table S2.pdf]

Table S2 Primer sequences for qPCR.

Gene	Primer sequence	
	**Forward**	**Reverse**
Il-6	5′-AGACAAAGCCAGAGTCCTTCAGAGA-3′	5′-GCCACTCCTTCTGTGACTCCAGC-3′
IL-1β	5′-GTTCTTAAGGTGTTCCTGGAGA-3′	5′-ATCCATTGCAGTGACTGTCTTA-3′
Tnf-a	5′-CCCTCCTGGCCAACGGCATG-3′	5′-CCCTCCTGGCCAACGGCATG-3′
Tgf-β	5′-CGGGAAGCAGTGCCCGAACC-3′	5′-GGGGGTCAGCAGCCGGTTAC-3′
Sod1	5′-GCGATGAAAGCGGTGTGCGTG-3′	5′-TGGACGTGGAACCCATGCTGG-3′
Gsh-rd	5′-GGGATGCCTATGTGAGCCGCC-3′	5′-TGACTTCCACCGTGGGCCGA-3′
Gsh-px	5′-GGTGGTGCTCGGTTTCCCGT-3′	5′-AATTGGGCTCAACCCGCCAC-3′
Cpt1	5′-CTCCGCCTGAGCCATGAAG-3′	5′-CACCAGTGATGATGCCATTCT-3′
Pgc-1α	5′-CCACTTCAATCCACCCAGAAAG-3′	5′-TATGGAGTGACATAGAGTGTGCT-3′
Srebp-1c	5′-GATGTGCGAACTGGACACAG-3′	5′-CATAGGGGGCGTCAAACAG-3′
Acc	5′-GCCCTTCTGAGTCGCTTAATATG-3′	5′-TGACATCACCCCTAGAGTCCT-3′
Fasn	5′-TATCAAGGAGGCCCATTTTGC-3′	5′-TGTTTCCACTTCTAAACCATGCT-3′
Nrf2	5′-TTTGTAGATGACCATGAGTCGC-3′	5′-TGTCCTGCTGTATGCTGCTT-3′
HO-1	5′-CTCCCAGGGCCATGAACTTT-3′	5′-GGGAAGATGCCATAGGCTCC-3′
GPX4	5′-ATTCCCGAGCCTTTCAACC-3′	5′-ACGCAACCCCTGTACTTATCC-3′
Gapdh	5′-AGGTCGGTGTGAACGGATTTG-3′	5′-TGTAGACCATGTAGTTGAGGTCA-3′
